# Supplementary figures and images for: Protective effects of clinical anticholinergic and anticholinesterase agents against Bungarus multicinctus venom and neurotoxin-rich snake venoms
Source: PLoS Negl Trop Dis. 2025 Dec 4;19(12):e0013759. doi: 10.1371/journal.pntd.0013759 (PMC12677460; doi:10.1371/journal.pntd.0013759)

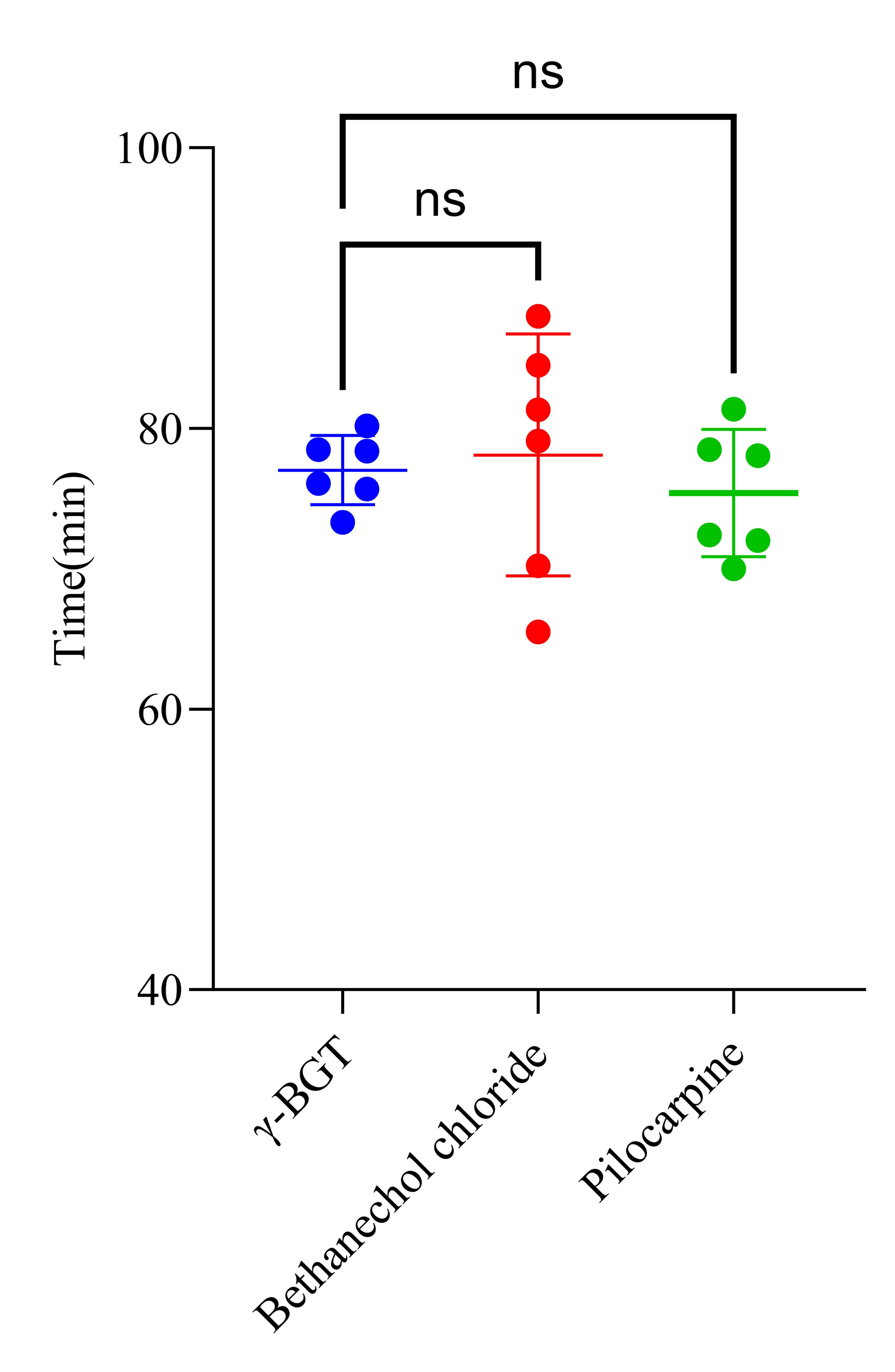

Supplement: S1 Fig — A: Protective effects of Bethanechol Chloride and Pilocarpine Hydrochloride against γ-bungarotoxin. Mice were pre-injected with the two drugs prior to γ-bungarotoxin administration; neither drug exerted a significant protective effect (p > 0.05, n = 6 per group). B: Protective effects of scopolamine and neostigmine methylsulfate against β-bungarotoxin. Mice were pre-injected with the two drugs prior to β-bungarotoxin administration; neither drug exerted a significant protective effect (p > 0.05, n = 6 per group). C: Protective effects of scopolamine and neostigmine methylsulfate against Ophiophagus hannah venom. Mice were pre-injected with the two drugs prior to O. hannah venom administration; neither drug exerted an effective protective effect (p > 0.05, n = 6 per group). (TIFF) [file pntd.0013759.s001.tiff]

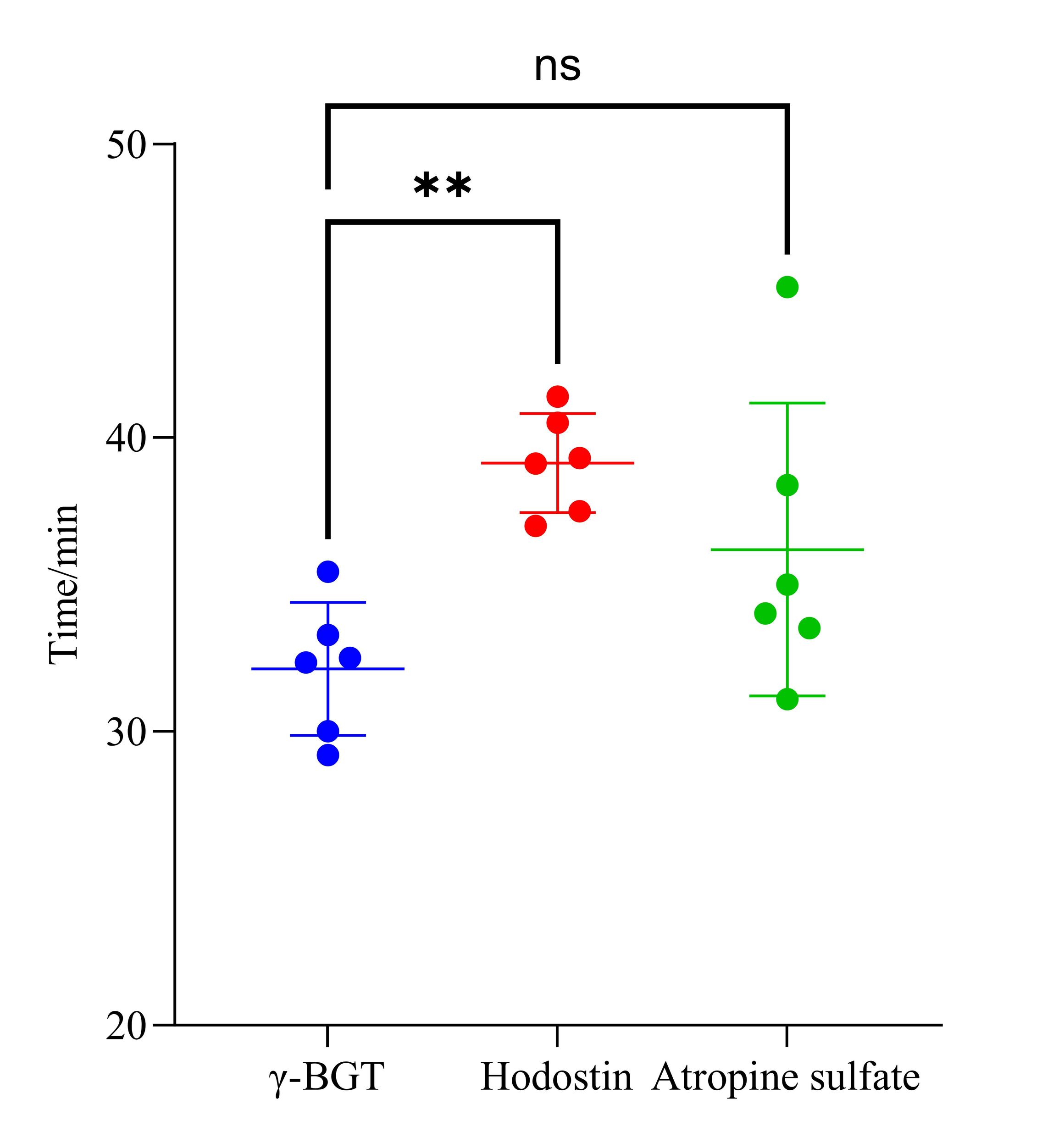

Supplement: S2 Fig — A: Protective effects of double-dose neostigmine methylsulfate and atropine sulfate against γ-bungarotoxin. After γ-bungarotoxin administration to mice, intravenous injection of double-dose atropine sulfate showed no significant effect (p > 0.05), while double-dose neostigmine methylsulfate accelerated mortality (p < 0.0001, n = 6 per group). B: Protective effects of neostigmine methylsulfate and atropine sulfate administered at 20 minutes post γ-bungarotoxin injection. Mice were administered the drugs via injection at 20 minutes after γ-bungarotoxin exposure: neostigmine methylsulfate still exerted a significant protective effect (p < 0.01), while atropine sulfate only slightly prolonged survival (p > 0.05, n = 6 per group). (TIFF) [file pntd.0013759.s002.tiff]

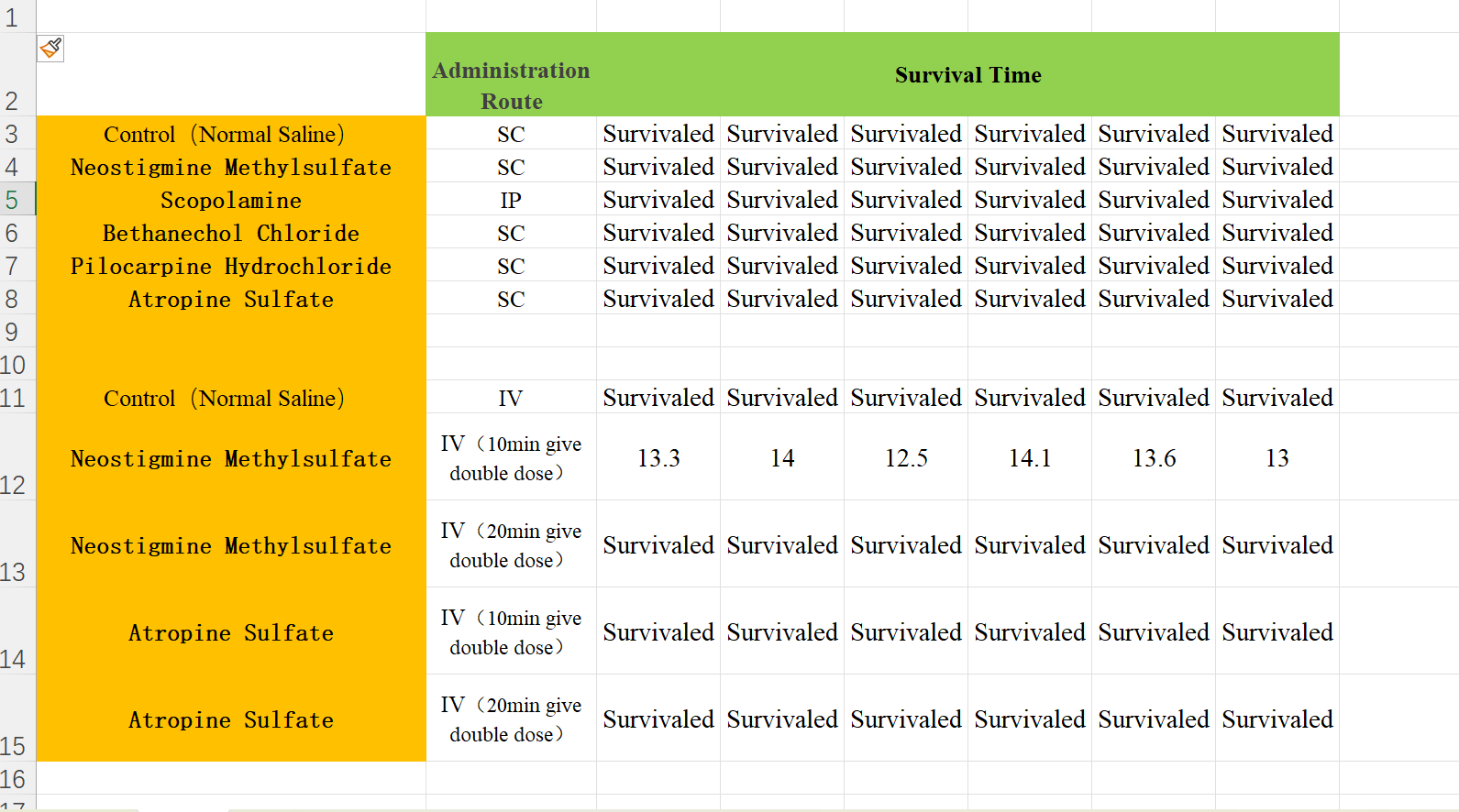

Supplement: S2 Table — This table summarizes the control groups corresponding to different drugs, injection routes, and injection timings. (PNG) [file pntd.0013759.s004.png]
