# Supplementary material for: Protective effects of clinical anticholinergic and anticholinesterase agents against Bungarus multicinctus venom and neurotoxin-rich snake venoms
Source: PLoS Negl Trop Dis. 2025 Dec 4;19(12):e0013759. doi: 10.1371/journal.pntd.0013759 (PMC12677460; doi:10.1371/journal.pntd.0013759)
Supplement: Attachment — Submitted filename: Response to reviewers.pdf [file pntd.0013759.s006.pdf]

Response to review's comments:

We sincerely extend our gratitude to the Editor and all Reviewers for their valuable comments and suggestions, which have significantly contributed to improving the quality of our manuscript. Below, we provide point-by-point responses to each comment raised by the Reviewers (shown in *italics*). Our replies are presented in normal font, while revisions and additions in the manuscript are highlighted in red.

Methods

1. *-Are the objectives of the study clearly articulated with a clear testable hypothesis stated?*

Responses: Thanks for your suggestions, the research objectives and hypotheses of this experiment have been presented in lines 19 to 22（Manuscript）in the Abstract section of the revised version.

2. *-Is the study design appropriate to address the stated objectives?*

Responses: Thanks for your suggestions, we believe the research design of this study effectively addresses the objectives and hypotheses we proposed, with a well-focused approach.

3. *-Is the population clearly described and appropriate for the hypothesis being tested?*

Responses: Thanks for your suggestions. This experiment utilized Kunming mice, with detailed specifications provided in the section 'Experimental Materials and Reagents—Ethical Statement'. The primary objective of this study was to investigate the protective effects of several anticholinergic or anticholinesterase agents against bungarotoxins venom and other neurotoxin-rich snake venoms. The key detection metric was survival time, which serves as an objective indicator. We are confident that the selected mice fully meets experimental requirements, and we believe the species choice has minimal impact on the research outcomes.

4. *-Is the sample size sufficient to ensure adequate power to address the hypothesis being tested?*

Responses: Thanks for your suggestions. The sample size for this experiment was set at six mice per group. Detailed information regarding sample size determination has been supplemented in the section 'Experimental Methods—Statistical Analysis'. This sample size was determined based on effect sizes reported in published studies with comparable experimental designs, with a minimum of n=6 per group to ensure adequate statistical power.

5. *-Were correct statistical analysis used to support conclusions?*

Responses: Thanks for your suggestions. The statistical methodology employed in this study has been comprehensively detailed in the section 'Experimental Methods—Statistical Analysis'. We are confident that this approach provides robust support for the final conclusions.

6. *-Are there concerns about ethical or regulatory requirements being met?*

Responses: Thanks for your suggestions. All animal experiments conducted in this study were in full compliance with applicable ethical and regulatory requirements. Comprehensive details have been documented in the section 'Experimental Materials and Reagents—Ethical Statement'. We extend our appreciation for your attention to this matter.

7. *Reviewer #1: The work is good and is presented well.*

Responses: Thanks for your suggestions. We sincerely appreciate the Reviewer's kind feedback and positive recognition regarding the quality of our work and its presentation. We are truly pleased to learn that our work has been well-received.

8. *Reviewer #2: More details of the assumptions on data and the statistical testing procedure should be provided.*

Responses: Thanks for your suggestions, The statistical methodology for this experiment has been detailed within the "Experimental Methods -- Statistical Analysis"

section as suggested.

9. *Reviewer #3: Objectives and Hypothesis: The study's objective-evaluating clinical agents as emergency treatments for B. multicinctus envenomation-is clearly stated. However, the hypothesis linking γ-bungarotoxin to severe clinical outcomes lacks explicit formulation.*

Responses: Thanks for your suggestions. We deleted the original description on γ -bungarotoxin. Current knowledge concerning γ -bungarotoxin were presented in lines 90-94 （Manuscript） in the revised version.

10. *Study Design: The preclinical mouse model appropriately tests toxin/drug interactions but omits critical design elements:*

(1) No randomization/blinding procedures described.

Responses: Thanks for your suggestions. The issue you rightly highlighted regarding the description of randomization and blinding procedures in the experimental design is very important. We have thoroughly reviewed this and provide the following clarifications.

Regarding Randomization:

We fully concur that the randomized allocation of experimental animals is critical for minimizing experimental bias. In fact, during the revision process, we recognized this point and have explicitly added a detailed description of the randomization method used for animal group assignment in Randomization of the Experimental Methods section.

Regarding Blinding Procedures:

We acknowledge the importance of blinding in experimental design, particularly in studies involving subjective assessments. However, after careful consideration, we determined that implementing operator or outcome assessor blinding was not deemed necessary for this specific experiment, for the following reasons:

(a) Objective Primary Endpoint: The primary endpoint of this study was Survival Time. This is a completely objective, fact-based endpoint. Its determination does not depend on the investigator's subjective judgment or expectation.

(b) Objectivity of Result Recording: Recording survival time constitutes a clear,

unambiguous objective event. The recorder cannot alter the actual time of death based on knowledge of group assignment. The data recording process itself is inherently resistant to the introduction of subjective bias.

(c) Nature as a Core Mechanistic Study: The primary objective of this study was to evaluate the protective effect of the drug against toxin-induced lethality. The core focus was on observing the objective outcome of survival/death, rather than on subjective assessments of symptom severity or behavioral changes.

Therefore, while we fully recognize the value of blinding in clinical trials or studies involving subjective endpoints, given the objective nature of the primary endpoint in this experiment, we concluded that implementing blinding was neither feasible nor a critical requirement for safeguarding the objectivity of the core result (survival time). The focus of the experimental design in ensuring result reliability was already addressed through stringent randomization and the use of an objective endpoint.

We sincerely appreciate your rigorous feedback, which prompted us to articulate the key aspects of our experimental design more clearly. We have supplemented the methodological details concerning randomization in the manuscript and have provided here the specific rationale for not employing blinding. We hope the above explanation adequately addresses your concerns.

(2) *Dosage selection (e.g., 0.05 mg/kg atropine) lacks pharmacokinetic justification for human translation.*

Responses: Thanks for your suggestions. The formula for drug dosage conversion between mice and humans used in this experiment has been detailed and further supplemented in the section "Experimental Methods -- Drug Dosage Calculation Formula".

(3) *Sample Size and Power: Absence of sample size calculation or power analysis undermines statistical validity. For example, crude venom experiments showing "modest survival prolongation" may be underpowered.*

Responses: Thanks for your suggestions. The statistical methodology for this experiment has been detailed and further supplemented within the "Experimental

Methods -- Statistical Analysis" section. Additionally, the sample size for this experiment was set at six mice per group. Detailed information regarding sample size determination has been supplemented in the section 'Experimental Methods—Statistical Analysis'. This sample size was determined based on effect sizes reported in published studies with comparable experimental designs, with a minimum of n=6 per group to ensure adequate statistical power.

(4) *Statistical Methods: Statistical tests (e.g., t-test vs. ANOVA) and software are unspecified. Reporting only P-values without effect sizes or confidence intervals limits interpretability.*

Responses: Thanks for your suggestions. The statistical methodology for this experiment has been detailed and further supplemented within the "Experimental Methods -- Statistical Analysis" section.

(5) *Ethical Compliance: Institutional Animal Care and Use Committee (IACUC) approval permit number is missing.*

Responses: Thanks for your suggestions. All animal experiments conducted in this study were in full compliance with applicable ethical and regulatory requirements. Comprehensive details have been documented in the section 'Experimental Materials and Reagents—Ethical Statement'. We extend our appreciation for your attention to this matter. (Approval ID: SMKX-2017023).

11. - *the study design appears appropriate to test the hypothesis, however there is a significant lack of detail in the methods section, making assessment and reproducibility difficult.*

Responses: Thanks for your suggestions. The original Methods section of the manuscript has been revised. Furthermore, content regarding randomization procedures, Drugs control groups, the drug dosage calculation formula, and statistical analysis has been supplemented. Please refer to the detailed descriptions in the sections: "Experimental Methods—Randomization", "Experimental Methods—Effects of Individual Drugs on Mice", "Experimental Methods—Drug Dosage Calculation Formula", and "Experimental Methods—Statistical Analysis"

12. *Furthermore, it isn't clear if there were control groups and the only outcome measured was survival, no morbidity outcomes.*

Responses: Thanks for your suggestions. Drug Control groups were established during the initial phase of this experiment. The detailed methodology has been described in the section "Experimental Methods—Effects of Individual Drugs on Mice" within the manuscript. The corresponding data are located in "Supporting Information—Table 1". Furthermore, the primary objective of this study is to investigate the protective effects of several anticholinergic or anticholinesterase drugs against *Bungarus multicinctus* venom and neurotoxin-rich snake venoms, with the primary indicator being "survival time"—an objective measure. Survival time is the most direct and clinically relevant indicator of successful intervention against lethality (WHO Guidelines for Snake Antivenom Testing, 2016). Given the rapid neuroparalytic action of α/β-bungarotoxins (accounting for >80% mortality in envenomation models), survival outcome remains the gold standard for quantifying protection efficacy in neurotoxic envenomation studies (Gutiérrez et al., Toxicon 2017).

13. *-there is no data analysis section in the methods and therefore no description of data analysis*

Responses: Thanks for your suggestions, Detailed information regarding the statistical methods employed in this study has been supplemented in the section "Experimental Methods—Statistical Analysis".

Results

1. *Reviewer #1: The results are clear*

Responses: Thanks for your suggestions, We sincerely appreciate the Reviewer's kind feedback and positive recognition regarding the quality of our work and its presentation. We are truly pleased to learn that our work has been well-received.

2. *Reviewer #2: The results can be organized better so that the major findings are*

clear. Graphs can be presented concisely to better compare the treatments with respective toxin combinations.

Responses: Thanks for your suggestions. We have revised the section entitled "Protective Effects of Pre-Administered Anticholinergic and Anticholinesterase Drugs against Different Snake Venoms". Results lacking statistical significance have been removed from the main text and relocated to Supporting Information (see Supporting Information Fig 1 and Fig 2). We believe the retained findings are sufficiently important to warrant inclusion in the main body of the manuscript.

Regarding the suggestion to consolidate several figures into one, we found this challenging to implement effectively. Due to inherent batch-to-batch variation observed in the experiments, resulting in differing survival times, such consolidation could compromise the clarity and visual interpretation of the results.

Similarly, while presenting the data in tabular form would offer greater conciseness, we are concerned it might obscure the clear visual representation of the key outcomes. However, should you deem it necessary to convert the figures to tables, we would be happy to implement this revision.

3. *Reviewer #3: Analysis Alignment: Results for purified toxins align with the methods, but crude venom experiments (showing non-significant effects) lack mechanistic follow-up to address this critical gap.*

Responses: Thanks for your suggestions. To address this question, we added "Although the neurotoxic symptoms were mainly attributed to β-bungarotoxin, the effects of other three-finger bungarotoxins contained in the venom should have their roles contributed in neurotoxic symptoms. Thus, neostigmine methylsulfate and atropine sulfate might have their value in certain conditions where victims bitten by *B. multicinctus* might benefit by administration these drugs before antivenom available." In the revised version, see lines 407-4112（Manuscript） in the resubmitted manuscript.

4. *Figures/Tables referenced in-text (e.g., Table 1, Figure 2) are absent from the submitted draft.*

Responses: Thanks for your suggestions. We have now thoroughly reorganized

all figures and tables throughout the manuscript. All referenced figures have been submitted as supplementary attachments alongside the draft, with comprehensive details provided in the documents titled "Charts and graphs in the article" and "Supporting Information". Furthermore, when we resubmit the revised draft, the corresponding figures and tables will be incorporated into their appropriate locations within the main text body.

5. *Survival curves for toxin combinations are described but not visualized.*

Responses: Thanks for your suggestions. We have now thoroughly re-organized all figures and tables throughout the manuscript. All referenced figures have been submitted as supplementary attachments alongside the draft, with comprehensive details provided in the documents titled "Charts and graphs in the article" and "Supporting Information". Furthermore, when we resubmit the revised draft, the corresponding figures and tables will be incorporated into their appropriate locations within the main text body.

6. *Statistical Reporting: Inconsistent reporting (e.g. \(P<0.01\) for toxins vs. \(P > 0.05\) for crude venom) requires full statistical detail for verification.*

Responses: Thanks for your suggestions. Detailed information regarding the statistical methods employed in this study has been supplemented in the section "Experimental Methods—Statistical Analysis". Furthermore Relevant p-values have been added to the appropriate locations within the Results section.

7. *Reviewer #4: - analysis plan wasn't described in the methods*

Responses: Thanks for your suggestions. Detailed information regarding the statistical methods employed in this study has been supplemented in the section "Experimental Methods—Statistical Analysis".

8. *- figures lack clear description and titles are too vague, difficult for the reader to understand what is what*

Responses: Thanks for your suggestions. The titles of the figure legends, Methods section, and Results section have been revised to provide more detailed and explicit

descriptions, enhancing clarity.

9. *-the results section has graphs, but doesn't contain the exact data and p-value (e.g. 61 mins vs.50 mins \(p=0.03\)) in the text, just saying "significant protection"*

Responses: Thanks for your suggestions. Relevant p-values have been added to the appropriate locations within the Results section. The specific survival times, however, have not been added to the main text at this stage for the following reason: these detailed temporal data are already presented in the figures and Supporting Information; we believe restating them in the main text would risk redundancy, as they remain readily accessible to readers.

10. *-also there are a lot of figures, I wonder if it might be more fitting in a table? Or combined figures?*

Responses: Thanks for your suggestions. We have revised the section entitled "Protective Effects of Pre-Administered Anticholinergic and Anticholinesterase Drugs against Different Snake Venoms". Results lacking statistical significance have been removed from the main text and relocated to Supporting Information (see Supporting Information Fig 1 and Fig 2). We believe the retained findings are sufficiently important to warrant inclusion in the main body of the manuscript.

Regarding the suggestion to consolidate several figures into one, we found this challenging to implement effectively. Due to inherent batch-to-batch variation observed in the experiments, resulting in differing survival times, such consolidation could compromise the clarity and visual interpretation of the results.

Similarly, while presenting the data in tabular form would offer greater conciseness, we are concerned it might obscure the clear visual representation of the key outcomes. However, should you deem it necessary to convert the figures to tables, we would be happy to implement this revision.

11. *- interpretation of results should be in the discussion section*

Responses: Thanks for your suggestions. The Results section has been revised accordingly. Furthermore, the interpretation of these results has been moved to the Discussion section.

12. *-is there a control group? If not, this is a limitation*

Responses: Thanks for your suggestions. Vehicle control groups (also referred to as Drug Control groups in this context) were established during the initial phase of this experiment. The detailed methodology has been described in the section "Experimental Methods—Effects of Individual Drugs on Mice" within the manuscript. The corresponding data are located in "Supporting Information—Table 1".

Conclusions

1. *Reviewer #1: Conclusions are well supported by the data presented*

Responses: Thanks for your suggestions. We sincerely appreciate the Reviewer's kind feedback and positive recognition regarding the quality of our work and its presentation. We are truly pleased to learn that our work has been well-received.

2. *Reviewer #2: Not needing Non-Parametric Statistical tests should be convinced to the reader before making conclusions.*

Responses: Thanks for your suggestions. Detailed information regarding the statistical methods employed in this study has been supplemented in the section "Experimental Methods—Statistical Analysis".

3. *γ-bungarotoxin was the primary focus of this study. However, the protective effects from Neostigmine methylsulfate and atropine sulfate is minimally discussed in the manuscript. Given the extensive data set more analysis can be done on this respect.*

Responses: Thanks for your suggestions. We deleted the original description on γ-bungarotoxin. Current knowledge concerning γ-bungarotoxin were presented in lines 90-94 in the revised version.

4. *Reviewer #3: Support from Data: Claims about clinical utility are overstated given the lack of efficacy against whole venom. The conclusion that drugs "provide supplementary benefits" is unsupported by crude venom data.*

Responses: Thanks for your suggestions. Since our results show weak efficacy against whole venom. We added “Thus, neostigmine methylsulfate and atropine sulfate might have their value in certain conditions where victims bitten by *B. multicinctus* might benefit by administration these drugs before antivenom available.” In the revised version.

5. *Limitations: Critical limitations are unaddressed:*

Responses: Thanks for your suggestions. Critical limitations were added at the end of discussion as suggested, see lines 450-452 of the revised version.

6. *Translational relevance of mouse-to-human dosing.*

Responses: Thanks for your suggestions, The formula for drug dosage conversion between mice and humans used in this experiment has been detailed and further supplemented in the section "Experimental Methods -- Drug Dosage Calculation Formula".

7. *Inability of drugs to neutralize crude venom despite toxin efficacy.*

Responses: Thanks for your suggestions. We noticed that other reviewers also pointed out this question. We carefully discussion this issue in the revised version.

8. *Public Health Relevance: The focus on remote settings is well-placed, but failure to demonstrate whole-venom efficacy reduces practical impact.*

Responses: Thanks for your suggestions. We added “Although the neurotoxic symptoms were mainly attributed to β-bungarotoxin, the effects of other three-finger bungarotoxins contained in the venom should have their roles contributed in neurotoxic symptoms. Thus, neostigmine methylsulfate and atropine sulfate might have their value in certain conditions where victims bitten by *B. multicinctus* might benefit by administration these drugs before antivenom available.” In the revised version.

9. *Reviewer #4: It is difficult to assess and interpret the results without the details of the study design and procedures.*

Responses: Thanks for your suggestions. We have revised sections including the Abstract, Experimental Materials and Reagents, and Experimental Methods to

incorporate previously omitted details. Key additions encompass:

- The research objectives and hypotheses
- Storage conditions for drugs and snake venoms
- Enhanced title completeness and readability
- Administration routes for each drug
- The injection site
- Randomization procedures
- Effects of Individual Drugs on Mice (drugs control groups)
- Drug dosage calculation formulae
- Statistical analysis methodologies

Furthermore, detailed protocols for different administration routes are provided below. These were not included in the main text considering they represent established methodologies where extensive description may unduly lengthen the manuscript. Should this be required, we would be pleased to supply them as supplementary material. We sincerely appreciate your attentive review.

（a） The subcutaneous injection procedure was performed as follows: After depilating the dorsal fur of mice, venom or drug solutions were loaded into disposable syringes. The skin was tented at the injection site, and the needle was inserted subcutaneously to an appropriate depth (approximately 5-10 mm) for substance administration.

（b） The intraperitoneal injection protocol was conducted as follows: Venom or drug solutions were aspirated into sterile disposable syringes. Mice were manually restrained in a supine position, and injections were performed in the lower abdominal quadrant midway between the hind limbs and ventral midline. Needles were inserted into the peritoneal cavity at a 15-30° angle to a depth of 5-8 mm for fluid delivery.

（c） The mouse tail vein injection protocol comprised the following steps: An appropriate insulin syringe (29-31 gauge) was loaded with venom or drug solution. Mice were secured in a dedicated restraint device. Tail veins were dilated by gentle warming and alcohol swabbing. The needle was inserted into the lateral tail vein at a 15-30° angle, followed by slow infusion of the substance over 5-10 seconds.

10. *Limitations are missing*

Responses: Thanks for your suggestions. Critical limitations were added at the end of discussion as suggested, see lines 450-452 of the revised version.

Editorial and Data Presentation Modifications

1. *Reviewer #1: The work is good and is presented well. But need some minor revisions*

Responses: Thanks for your suggestions, We sincerely appreciate your favorable comments on our experimental work and your recognition of its contributions. We are also truly grateful for your insightful observations regarding the issues requiring attention.

（1） *Kindly spell check the entire document. Even snake was misspelled as 'snak' in keywords.*

Responses: Thanks for your suggestions, We have thoroughly reviewed the entire manuscript and corrected the spelling errors identified. Thank you for bringing these matters to our attention.

（2） *Most of the paragraphs starts with 'To...', which is usually not allowed in research papers.*

Responses: Thanks for your suggestions, We have carefully reviewed the entire manuscript, with particular attention to revising paragraphs beginning with infinitives (e.g., "To investigate..."). Thank you for bringing this observation to our attention.

2. *Reviewer #3: Data Availability: Statement must specify repository (e.g., Dryad/Zenodo) and accession DOI per PLOS policy.*

Responses: Thanks for your suggestions. We attempted to deposit the data into relevant repositories, including platforms such as Dryad or Zenodo as you suggested. However, most repositories incur fees for data submission; for example, Dryad charges

a fee of $150 per submission . Additionally, due to potential regional access issues, we encountered difficulties accessing certain repositories like Zenodo, which might be geographically restricted . Given these considerations, we deem it more appropriate to submit all study data as supplementary files alongside the manuscript. This approach ensures that all data remain publicly accessible while adhering to practical constraints . Thank you for the reminder, which has guided us toward a balanced solution.

3. *Figures/Tables: All referenced items must be included with clear legends.*

Responses: Thanks for your suggestions, We have conducted a comprehensive review and revision of the results section, addressing a range of improvements including but not limited to: reorganizing figures and tables, enhancing their visual clarity, and revising/expanding figure captions, Thank you for the reminder.

4. *Dosage Rationale: Add a table comparing mouse doses to human clinical ranges.*

Responses: Thanks for your suggestions, We have compiled a table to illustrate and compare mouse doses with human clinical dose ranges, which has been submitted as "Supporting Information--Mouse-to-Human Dose Conversion" . Given that human clinical dose ranges may vary due to regional variations and differences in clinical practices, this table is not included in the main text but provided as supplementary material, Thank you for the reminder.

5. *Mechanistic Clarification: Use subsections in Results to distinguish toxin-specific vs. venom-wide outcomes.*

Responses: Thanks for your suggestions. We carefully re-organized our manuscript and put some results in Supporting Information.

6. *Reviewer #4: The manuscript is generally written in understandable English, but there are some minor issues such as typos, incorrectly and overused used colons and incorrect capitalization. Also, line numbers are missing from the manuscript, making it difficult for reviewers to give exact examples.*

I would recommend the following:

Introduction

（1） *-adding some information about local burden of snake bites (morbidity and mortality), also are there DALYs for snake bites?*

Responses: Thanks for your suggestions. We added information in the introduction as suggested.

（2） *-giving background regarding current state of evidence for the use of these drugs for snake bites as well as current global and local recommendations/guidelines for the use of anticholinergic/anticholinesterase drugs in snake envenomation*

Responses: Thanks for your suggestions. We added information in the introduction and discussion sections as suggested.

Methods

（3） *The methods require more detail in order for readers to assess the methods, understand the results and for reproducibility. The details of the study design and procedures are not well described.*

Responses: Thanks for your suggestions. We have made detailed modifications and additions to the methodology and other sections of the manuscript, including the Abstract, Experimental Materials and Reagents, and Experimental Methods to incorporate previously omitted details. Key additions encompass:

- The research objectives and hypotheses
- Storage conditions for drugs and snake venoms
- Enhanced title completeness and readability
- Administration routes for each drug
- The injection site
- Randomization procedures
- Effects of Individual Drugs on Mice (drugs control groups)
- Drug dosage calculation formulae
- Statistical analysis methodologies

Furthermore, detailed protocols for different administration routes are provided below. These were not included in the main text considering they represent established methodologies where extensive description may unduly lengthen the manuscript.

Should this be required, we would be pleased to supply them as supplementary material. We sincerely appreciate your attentive review.

（4） *- Manufacturer and location of headquarters should be mentioned for materials (venom and drugs)*

Responses: Thanks for your suggestions. The sources of snake venom, bungarotoxins, drugs, and bungarus antivenom have been comprehensively revised and supplemented in the section titled **'Experimental Materials and Reagents — Venoms, Drugs and Antivenoms'**, as requested.

（5） *- I would recommend making the subtitles about the studies more succinct, maybe something like: Pre-exposure (or maybe "prophylactic anticholinergic and anticholinesterase"?) anticholinergic and anticholinesterase drugs, Post- exposure anticholinergic and anticholinesterase drugs and Delayed post-exposure anticholinergic and anticholinesterase drugs*

Responses: Thanks for your suggestions.In response to the valuable suggestions from you and the other reviewers, we have carefully revised the headings in both the *Methods* and *Results* sections to ensure clarity and completeness, as recommended.

（6） *- Section "Protective effects study of pre-administered bungarotoxins needs work, sentences are not complete?*

Responses: Thanks for your suggestions. We have made revisions and supplements to the titles and figure captions in the methodology and results sections.

（7） *- Is it appropriate to only measure time of survival? What about other effects of venom like hemorrhage? This should be discussed in the discussion as a limitation*

Responses: Thanks for your suggestions. We sincerely appreciate the reviewer's valuable suggestion regarding additional measurement indicators. We fully acknowledge that comprehensive endpoints (such as hemorrhage, myotoxicity, or neurobehavioral symptoms) could provide further mechanistic insights into snake venom pathology. However, our experimental design intentionally focused on survival time as the primary endpoint for the following scientifically justified reasons:

Alignment with Core Research Objectives: The primary objective of this study is to investigate the protective effects of several anticholinergic or anticholinesterase drugs against Bungarus multicinctus venom and neurotoxin-rich snake venoms. Survival time is the most direct and clinically relevant indicator of successful intervention against lethality (WHO Guidelines for Snake Antivenom Testing, 2016). Given the rapid neuroparalytic action of α/β-bungarotoxins (accounting for >80% mortality in envenomation models), survival outcome remains the gold standard for quantifying protection efficacy in neurotoxic envenomation studies (Gutiérrez et al., Toxicon 2017).

Pathophysiological Relevance to Tested Venoms: *B. multicinctus* venom is characterized by predominant presynaptic (β-bungarotoxin) and postsynaptic (α-bungarotoxin) neurotoxins, with minimal hemorrhagic/metalloproteinase activity. Hemorrhage is not a hallmark of krait envenomation; thus, measuring hemorrhagic endpoints would be physiologically misaligned with the venom's primary lethal mechanism. Our toxin selection (crude venom, purified neurotoxins, neurotoxin-rich venoms) specifically targets neurotoxicity-driven mortality.

（8） *-More information needed regarding the procedures:*

Responses: Thanks for your suggestions. We have revised sections including the Abstract, Experimental Materials and Reagents, and Experimental Methods to incorporate previously omitted details. Key additions encompass:

- The research objectives and hypotheses
- Storage conditions for drugs and snake venoms
- Enhanced title completeness and readability
- Administration routes for each drug
- The injection site
- Randomization procedures
- Effects of Individual Drugs on Mice (drugs control groups)
- Drug dosage calculation formulae
- Statistical analysis methodologies

Furthermore, detailed protocols for different administration routes are provided

below. These were not included in the main text considering they represent established methodologies where extensive description may unduly lengthen the manuscript. Should this be required, we would be pleased to supply them as supplementary material. We sincerely appreciate your attentive review.

（9） *how was time of survival measured? What outcomes?*

Responses: We thank the reviewer for this critical methodological clarification. The measurement protocols for survival time and outcome definitions were rigorously implemented as follows:

Survival time was defined as the interval (in minutes) from the completion of venom injection to the death of mice, measured accurately using a digital timer, with survival time serving as the primary outcome measure.

（10） *A data analysis section should be included.*

Responses: Thanks for your suggestions. Detailed information regarding the statistical methods employed in this study has been supplemented in the section "Experimental Methods—Statistical Analysis".

（11） *What were the exact procedures, e.g. groups studied? For example in the third group, were there two groups of 6 mice, one treated with neostigmine methylsulfate and one with atropine sulfate? Where were the SC injections injected, the abdomen? How were the venoms/drugs stored? Which drugs IV, which SC and which IM, what was the process for IV admin for example. Why were some drugs IV and some IP? What were the procedures for each group?*

Responses: Thanks for your suggestions. Yes, in the third group, each of the two subgroups contained 6 mice: one subgroup was treated with Neostigmine Methyl Sulfate, and the other with Atropine Sulfate. The sample sizes of all other experimental groups were consistent with this configuration. The subcutaneous injection site was specified as the lower dorsal region of mice, and this detail has been added to the first section of the "Experimental Methods" section. Both snake venom and drugs are stored in the -20°C compartment of the laboratory freezer, with supplementary details provided in the "Venoms, Drugs and Antivenoms" section. The administration methods

for different drugs have been revised and expanded in the "Experimental Methods" section. These methods were selected based on clinical administration practices and experimental objectives, and the detailed procedures for each method are outlined below in this response.

Specifically: We have made detailed modifications and additions to the methodology and other sections of the manuscript, including the Abstract, Experimental Materials and Reagents, and Experimental Methods to incorporate previously omitted details. Key additions encompass:

- The research objectives and hypotheses
- Storage conditions for drugs and snake venoms
- Enhanced title completeness and readability
- Administration routes for each drug
- The injection site
- Randomization procedures
- Effects of Individual Drugs on Mice (drugs control groups)
- Drug dosage calculation formulae
- Statistical analysis methodologies

Furthermore, detailed protocols for different administration routes are provided below. These were not included in the main text considering they represent established methodologies where extensive description may unduly lengthen the manuscript. Should this be required, we would be pleased to supply them as supplementary material. We sincerely appreciate your attentive review.

（a） The subcutaneous injection procedure was performed as follows: After depilating the dorsal fur of mice, venom or drug solutions were loaded into disposable syringes. The skin was tented at the injection site, and the needle was inserted subcutaneously to an appropriate depth (approximately 5-10 mm) for substance administration.

（b） The intraperitoneal injection protocol was conducted as follows: Venom or drug solutions were aspirated into sterile disposable syringes. Mice were manually restrained in a supine position, and injections were performed in the lower abdominal

quadrant midway between the hind limbs and ventral midline. Needles were inserted into the peritoneal cavity at a 15-30° angle to a depth of 5-8 mm for fluid delivery.

（c） The mouse tail vein injection protocol comprised the following steps: An appropriate insulin syringe (29-31 gauge) was loaded with venom or drug solution. Mice were secured in a dedicated restraint device. Tail veins were dilated by gentle warming and alcohol swabbing. The needle was inserted into the lateral tail vein at a 15-30° angle, followed by slow infusion of the substance over 5-10 seconds.

Summary and General Comments

1. *Reviewer #1: The work is good and is presented well. But need some minor revisions*

Responses: Thanks for your suggestions. We sincerely appreciate your favorable comments on our experimental work and your recognition of its contributions. We are also truly grateful for your insightful observations regarding the issues requiring attention.

2. *Kindly spell check the entire document. Even snake was misspelled as 'snak' in keywords.*

Responses: Thanks for your suggestions, We have thoroughly reviewed the entire manuscript and corrected the spelling errors identified. Thank you for bringing these matters to our attention.

3. *Most of the paragraphs starts with 'To...', which is usually not allowed in research papers.*

Responses: Thanks for your suggestions, We have carefully reviewed the entire manuscript, with particular attention to revising paragraphs beginning with infinitives (e.g., "To investigate..."). Thank you for bringing this observation to our attention.

4. *Reviewer #2: The literature review can be elaborated more.*

Responses: Thanks for your suggestions. We added some literatures in the revised

version as suggested.

5.*Results can be further discussed by relating the results to Human Physiological conditions.*

Responses: Thanks for your suggestions. We discussed this issue in the revised version.

6.*Reviewer #3: Strengths:*

Clinically significant focus on accessible emergency treatments for resource-limited regions. Rigorous evaluation of drug-toxin interactions with clear implications for adjunct therapy.

Responses: Thanks for your suggestions. We sincerely appreciate the Reviewer's kind feedback and positive recognition regarding the quality of our work and its presentation. We are truly pleased to learn that our work has been well-received.

Weaknesses Requiring Major Revision:

7.Statistical Rigor: Specify tests, software, and effect sizes; justify sample sizes.

Responses: Thanks for your suggestions. The statistical methodology for this experiment has been detailed and further supplemented within the "Experimental Methods -- Statistical Analysis" section. Additionally, Detailed information regarding sample size determination has been supplemented in the section 'Experimental Methods—Statistical Analysis'. This sample size was determined based on effect sizes reported in published studies with comparable experimental designs, with a minimum of n=6 per group to ensure adequate statistical power.

8.*Translational Gap: Correlate mouse dosages to human safety/efficacy thresholds.*

Responses: Thanks for your suggestions. We have compiled a table to illustrate and compare mouse doses with human clinical dose ranges (Effective-to-Safety Dose Range), which has been submitted as "Supporting Information--Mouse-to-Human Dose Conversion" . Given that human clinical dose ranges may vary due to regional

variations and differences in clinical practices, this table is not included in the main text but provided as supplementary material, Thank you for the reminder.

9.*Crude Venom Efficacy: Address non-significance via:*

Synergistic drug combinations.

Pre-treatment experimental designs.

Responses: Thanks for your suggestions. We carefully revised our manuscript and pay attention to above mention points.

10.*Ethical Compliance: Provide IACUC permit number.*

Responses: Thanks for your suggestions. All animal experiments conducted in this study were in full compliance with applicable ethical and regulatory requirements. Comprehensive details have been documented in the section 'Experimental Materials and Reagents—Ethical Statement'. We extend our appreciation for your attention to this matter. (Approval ID: SMKX-2017023).

11.*Novelty and Impact: The repurposing of FDA-approved drugs is innovative and aligns with PLOS NTDs' scope for practical solutions in tropical medicine. With revisions, this work could significantly impact snakebite management protocols.*

Responses: Thanks for your suggestions. We sincerely appreciate your favorable comments on our experimental work and your recognition of its contributions. We are also truly grateful for your insightful observations regarding the issues requiring attention.

12.*Reviewer #4: This is an interesting investigation into the effects of anticholinergic/anticholinesterase drugs pre- and post- snake envenomation. As an official NTD, it is helpful to have more information on management of morbidity and mortality due to snake bites.*

Responses: Thanks for your suggestions. We sincerely appreciate your favorable comments on our experimental work and your recognition of its contributions. We are also truly grateful for your insightful observations regarding the issues requiring attention.

13.*The manuscript requires work to be suitable for publication, especially in describing the study design and procedures in the methods. Furthermore, the study does need to be placed in context in the introduction, as it seems there have already been studies on the use of anticholinergic/anticholinesterase drugs in snake envenomation and there are guidelines about their use for this purpose (https://pmc.ncbi.nlm.nih.gov/articles/PMC11402871/).*

Responses: Thanks for your suggestions. We have thoroughly revised and supplemented the Methods section, particularly enhancing the descriptions of technical details and procedural steps.

14. *Figure resubmission:*

While revising your submission, please upload your figure files to the Preflight Analysis and Conversion Engine (PACE) digital diagnostic tool, https://pacev2.apexcovantage.com/. PACE helps ensure that figures meet PLOS requirements. To use PACE, you must first register as a user. Registration is free. Then, login and navigate to the UPLOAD tab, where you will find detailed instructions on how to use the tool. If you encounter any issues or have any questions when using PACE, please email PLOS at figures@plos.org. Please note that Supporting Information files do not need this step. If there are other versions of figure files still present in your submission file inventory at resubmission, please replace them with the PACE-processed versions.

Responses: Thanks for your suggestions. We have reprocessed all figures through the **P**reflight **A**nalysis and **C**onversion **E**ngine (PACE) and replaced the original versions. Upon submission of the revised manuscript, both the main article files ('Charts and Graphs in the Article') and supplementary materials ('Supporting Information') containing the updated figures will be resubmitted. We greatly appreciate your guidance on this matter.

15.Reproducibility:

To enhance the reproducibility of your results, we recommend that authors of applicable studies deposit laboratory protocols in protocols.io, where a protocol can be

assigned its own identifier (DOI) such that it can be cited independently in the future. Additionally, PLOS ONE offers an option to publish peer-reviewed clinical study protocols. Read more information on sharing protocols at https://plos.org/protocols?utm_medium=editorialemail&utm_source=authorletters&utm_campaign=protocols

Responses: Thanks for your suggestions. As requested, we have deposited the experimental protocols for this study on *protocols.io*. The protocol is publicly accessible at: https://www.protocols.io/edit/protective-effects-of-clinical-anticholinergic-and-g658bzg9x with the assigned Digital Object Identifier (DOI): https://doi.org/10.17504/protocols.io.yxmvmb2nog3p/v1. We sincerely appreciate your guidance in enhancing the reproducibility of our research.
