# Supplementary material for: Protective effects of clinical anticholinergic and anticholinesterase agents against Bungarus multicinctus venom and neurotoxin-rich snake venoms
Source: PLoS Negl Trop Dis. 2025 Dec 4;19(12):e0013759. doi: 10.1371/journal.pntd.0013759 (PMC12677460; doi:10.1371/journal.pntd.0013759)
Supplement: Attachment — Submitted filename: Response to Reviewers-R2.docx [file pntd.0013759.s007.docx]

Reviewers' Comments:

Reviewer #3: The study employs well-defined animal models with clear descriptions of drug and venom administration to evaluate protective effects. Randomization by body weight is a strength that reduces bias. Statistical analyses are appropriate, and ethical standards are met. However, the use of high venom doses (2× or 3× LD₅₀) may produce overly severe effects that do not fully replicate real snakebite conditions. Additionally, the rationale for the selected time intervals between drug and venom injections is not adequately justified, limiting the clinical relevance of the findings. Overall, while the methodology is sound, providing justification for dose selection and timing would enhance the study’s translational applicability.

**Responses:** Thanks for your suggestions. We do agree that the use of high venom doses (2× or 3× LD₅₀) may produce overly severe effects that do not fully replicate real snakebite conditions. However, in animal rescue experiments, normally the venom dose used is 3× LD₅₀, like a recent published paper (Nature. 2025 Oct 29. doi: 10.1038/s41586-025-09661-0. Titled “Nanobody-based recombinant antivenom for cobra, mamba and rinkhals bites”. This paper reported in the rescue experiments, 3 × LD_50_ of each of the selected venoms were first injected and the recombinant antivenom was injected 5 min later). We added “the selected dosage is benefit to evaluate the effects of the drugs because the higher dosage make the tested animals dead quickly” (lane 160-161). We also added “In vivo protection of venom-induced lethality assays were mainly according to the methods described by Ahmadi et al.” in the revised version of the manuscript, see lane 169-170. The reason for the selected time intervals between drug and venom injections is based on the death time of only venom group and the time intervals of the drugs after venom injections were approximately 1/4 of the death time which might be mimic clinical relevance of the snake-bite situations. We also add this in the revised manuscript, see lane 179-182.

Reviewer #2: (No Response)

Reviewer #3: The results follow the planned analysis and clearly show the effects of the drugs tested. The use of survival times is straightforward, but adding other measures could strengthen the conclusions. The figures are clear, but referring too much to supplementary data makes it a bit hard to follow. Overall, the results are well presented but could be easier to understand with fewer cross-references.

**Responses:** Thanks for your suggestions. In the first version of our manuscript, one of Reviewer pointed out that *“The results can be organized better so that the major findings are clear. Graphs can be presented concisely to better compare the treatments with respective toxin combinations.”* Thus, results lacking statistical significance have been removed from the main text and relocated to Supporting Information. According to your suggestions, we added Fig. 2E and 2F to show the protective effects of neostigmine methylsulfate and atropine sulfate on pre-administration of *B. multicinctus* and *N. atra* crude venom, respectively.We hope it is acceptable for the revise manuscript.

Reviewer #2: (No Response)

Reviewer #3: The conclusions are well supported by the data, showing certain drugs protect against *B. multicinctus* venom. The authors clearly mention limitations, such as the drugs’ negative effects on other snake venoms. They explain how these findings improve understanding of snakebite treatment, especially when antivenom is not immediately available. The study also highlights important public health issues for rural areas lacking quick access to antivenom.

**Responses:** Thanks for your positive comments.

Reviewer #2: (No Response)

Reviewer #3: The Methods and Results sections are generally clear and well-structured, providing detailed descriptions and relevant data. To enhance clarity, it’s recommended to briefly justify key experimental choices such as venom doses and timing of drug administration in Methods. In Results, ensure all figures and tables are clearly labeled with consistent terminology and units. Adding brief explanations of statistical tests used would strengthen transparency. Simplifying complex sentences and avoiding jargon will improve readability. Overall, minor editorial refinements here will make the sections more accessible and scientifically robust.

**Responses:** Thanks for your suggestions. Key experimental choices such as venom doses and timing of drug administration in Methods were described as suggested, this point also pointed out by reviewer 3 and we carefully revised, see resubmitted manuscpirt. Other parts of the mentioned points were carefully checked and corrected. See revised version.

Reviewer #2: (No Response)

Reviewer #3: (No Response)
